# Safety culture influence on safety performance of a post-combustion carbon capture facility

**DOI:** 10.1016/j.heliyon.2024.e34640

**Published:** 2024-07-18

**Authors:** Maryam Shourideh, Sirous Yasseri, Hamid Bahai

**Affiliations:** a Mechanical Engineering, Brunel University London, United Kingdom; b Institute Director Materials & Manufacturing, Brunel University London, United Kingdom

**Keywords:** Safety Culture, Safety performance, System dynamics, Post combustion carbon capture facility, Safety policy

## Abstract

This article explores the influence of safety culture (as a subset of organizational culture) on the safety performance of a post-combustion carbon capture facility. After determining the controlling variables of safety culture, a system dynamics model was built to assess how those variables contribute to the safety performance of the facility. The focus on safety culture arises for avoiding major disasters that could significantly impact a company's ability to continue, as well as minor but disruptive incidents occurring during routine operations (i.e. when there is no system upset). This paper describes the complex relationship between cultural norms, leadership practices, communication patterns, and safety conduct with an emphasis on management and personnel commitment to safety, open communication, safety investments, and productivity pressure. Insights from this study contribute to the development of strategies for enhancing the safety performance of carbon capture operations, thereby promoting the integrity and reliability of these essential elements of energy networks. This paper focuses on the visible aspect of safety culture as manifested in organismal practices. We proposed a system dynamics model to devise strategies to reconcile the profitability while preventing accidents.

## 1 Introduction

For carbon Capture technology to take hold, it must be profitable, safe, and flexible. It can be bolted onto existing plants or new builds without requiring much modification of the energy-generating system. The affordability of carbon capture and storage (CCS) poses a significant challenge to the technology's success, leading to difficulties in maintaining safety systems when cost reduction is necessary for project feasibility. Considering the public hesitations regarding operational safety, organizations in high-risk sectors, such as nuclear, are obliged to address public concerns. In industries focused on cost-effectiveness, the pressure felt by organizations is centered on maintaining profitability and thus survival. As a result, organizations direct their focus and allocate safety budgets based on the demands of the public and regulator, while remaining solvent. There is also regulatory pressure to ensure the safety and security of the CCS installations [1].

Management's behaviors and priorities have the greatest influence on safety-related engineering issues since they set targets and policies as well as oversee their enforcement. Organizations must cultivate a strong safety culture combining procedural and engineering approaches to tackle challenges effectively. Simply investing in safety measures does not guarantee desired outcomes. Management is essential in motivating employees to align with organizational goals, despite occasional conflicts. Poor return on investment and insufficient safety performance can be disadvantageous. Safety protocols aim to prevent incidents.^1^ However, as incidents decrease, the urge to cut safety budgets could have lasting negative effects. Safety systems depend on collective organizational commitment, not just one individual or short-term campaign. Building employee relationships, developing trust, and establishing effective communication channels take time to ensure thorough understanding and consistent adherence to company protocols.

Any system is a collection of equipment, people, and its environment. These elements interact through the linkages that connect them [2,3]. The arrangement of elements within the system follows a scheme that enables the system to function. The arrangement of all elements of the system and the feedback between interacting components govern the system's functionality.

System Dynamics (SD) methods can be a valuable tool for understanding how various system components interact and influence each other. This paper uses SD modeling to investigate the study in what ways safety performance is influenced by safety culture.

Establishing a strong safety culture is critical for the success and sustainability of post-combustion carbon capture facilities. By adopting an environment where safety is embedded in all operational facets, these facilities can function effectively, keep their employees and the environment safe, and play a responsible role in global climate change mitigation actions. Safety culture exceeds simple regulatory obligations; it is a core element in securing the success and sustainability of post-combustion carbon capture facilities.

**Fig. 1:**
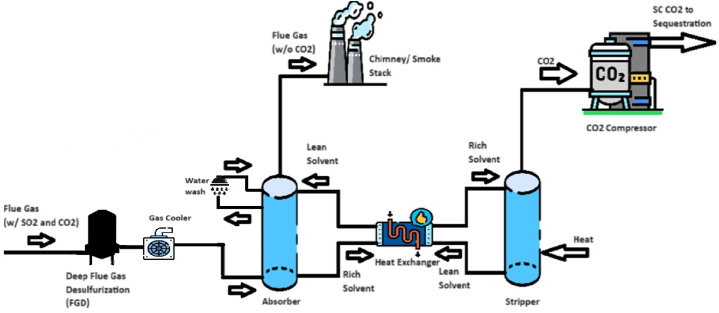
The general arrangement of a post-combustion carbon capture facility.

One of several carbon capture technologies employs a mix of organic amine solvents and water for absorbing carbon dioxide. This technology can be employed for various CO2-emitting sources, including gas or coal power stations, cement, refineries, and waste-to-energy by producing hydrogen. The absorption of CO2 requires a close relationship between the design of the solvent and the process configuration, resulting in a service that leads to high capture with minimal energy consumption and minimal degradation [4]. Fig. 1 shows an artist's impression of a typical post-combustion carbon capture plant.

This paper considers a post-combustion carbon capture facility with a track record as an exemplar. The CCS technology must prove itself as a viable technology on many dimensions, such as public acceptance, Capital Expenditure (CAPX), Operating Expenditure (OPEX), profitability, and of course safety. High-reliability requirements of carbon capture facilities are expected, which makes it a complex system. The carbon capture chain is a socio-technical system, consisting of several subsystems. This makes the recovery from any failure difficult; thus, the consequence of failure would not remain within the boundaries of the facility. Major industrial accidents^2^ have raised awareness of the contribution of human errors, as a result leading to the blame culture. Recently the attention has shifted from human contribution to failures as the single source of accidents, to the consequence of interacting technical issues and human elements [[5], [6], [7]].

The concept of system safety has been interpreted in various manners by numerous scientific and technical experts. Nonetheless, from its beginning, system safety has consistently aimed to eliminate system faults, failure risks, and potential accidents or hazards by incorporating engineering controls into the design and implementation processes [8].

## 2 Materials and methods

### 2.1 Safety Culture

Cultures are defined by shared values and beliefs, procedures, and practices, which are the ways personnel respond to events [9]. Many subcultures are denied for an organisation and the organisation culture is the highest level of hierarchy. Safety Culture is a subset which ensures these are aligned for safe operation. Organizations are a mosaic of different and complex cultures, with a multitude of assumptions, values, beliefs, and accepted collective behaviour. Thus, safety culture is viewed as a subsystem of the organisational culture whose structure is shaped by non-verbalised organisational practices. Generally, the first line of defence in any technical system is instrumented safety devices as well as process safety instruments, whose availability depends on operators to keep them in working condition. It is not uncommon for the human operator to disable part of the instrumented safety system, leave sensors in a disrepair state, or not check them periodically if they deliver the required safety functions. A tight budget, lack of time and profitability pressure could aggravate the problem. People who set production policies and enforce them are far from those who maintain and operate a system. From basic audits to continuous improvement, often organizations require cultural shifts and long-term vision which are difficult to manage. Understanding safety culture is necessary for safety improvement protocols. However, a basic question is, what safety culture is in a socio-technical system? What are the influencing factors that impact safety performance and how to influence safety culture to achieve desirable outcomes?

**Fig. 2:**
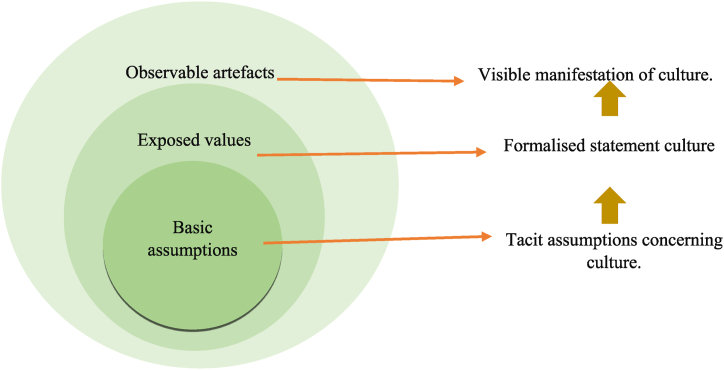
Schein's nested model of organisational culture [10].

Schein stated “Organizational culture can be thought of as consisting of three nested layers” as shown in Fig. 2 [10]. The inner layer (or core) comprises its fundamental assumptions and beliefs: what people value; what contributes to performance; what performance means; and the stories members relate to new people joining the team. These are intangible, tacit (not verbalised or written), and unspoken attitudes and beliefs (personnel's assumptions). The middle layer consists of values, shared principles, rituals, behavioural standards, and goals. It also includes mission statements about the organization's values and rules of conduct (how the members make sense of the organization to themselves and represent it to others). The outer layer includes artefacts, the physical environment, interface & interaction mechanisms, official policies, the dress code, and other visible parts of how people in the organization interact with each other [10].

**Fig. 3:**
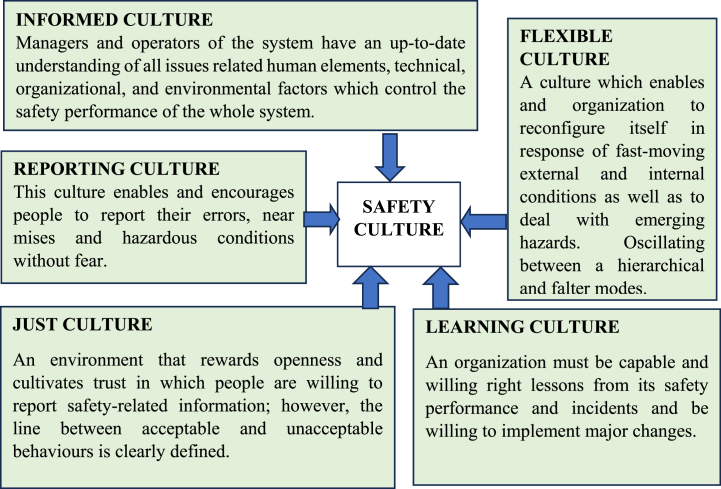
Reason's aspects of organizational culture [11].

Schein [10] has also identified three subgroups that exist in many organizations: executives, whose primary concern is financial performance; engineers, who deal with technical issues, and workers, who follow procedures [10]. In complex organizations, there are many other subgroups in parallel with the above subgroups based on ethnicity, spoken language, age, occupation, seniority, shift, and previous occupation [12]. People may be part of several subgroups, but one is generally dominant. Another groping of behaviours is proposed in the literature, For example, Reason has identified various aspects of safety culture which are shown in Fig. 3. In this paper, these are termed dimensions of safety culture, also referred to as variables influencing the safety culture [11]. Table 1 shows a few examples of the safety culture definition (chronologically) in the literature. There are many more definitions for safety culture.

**Table 1:** Examples of the safety culture definition (from various sources).

Definition	Quoted from
Safety climate, a subset of organizational climate, mirrors employees' views on the significance of safety in their work behaviour. It ranges from highly positive to neutral, with the average indicating the safety climate within each company.	[13]
Safety culture refers to the collective organisational values and beliefs that shape organizational behaviour and establish rules of conduct.	[14]
The interplay of roles, attitudes, norms, beliefs, and practices by employees trying to control risks to themselves, customers, the public, and the organisation in performing their duties, by avoiding hazardous practices.	[15]
Safety culture is the collection of attributes and mindsets within organizations and individuals that emphasize the utmost importance of giving due attention to nuclear plant safety issues due to their critical significance.	[16]
Safety culture encompasses the collective actions, values and beliefs held by all organizational hierarchies at all levels regarding risk, accidents, and health issues.	[17]
Safety culture emerges from the merging of individual values with the collective values, beliefs, skills, and behaviours that impact the visible aspect of an organization's behaviour regarding health, safety, and environment (HSE) issues. The characteristics of organizations that enforce a pragmatics safety culture are open communication based on mutual trust, no blame, a common understanding of the significance of safety, and value the of precautionary measures to avoid harm.	[18]
The safety climate represents the outward expressions of the safety culture, which originate from the perceptions, and perception of the workforce.	[19]
Safety climate is the result of employees' perceptions, and attitudes regarding the existing safety efforts in their workplace.	[20]

The above definitions share similarities as they can be classified from the perspective of normative beliefs [21]. They emphasize how people think, decide, and behave regarding practices as they relate to safety. Cox and Cox noted the broadness of the definitions given in Table 1, weakens their usefulness [22]. They suggested a much crisper and tighter definition is necessary [22]. To ensure clarity, the definition set by the ‘Advisory Committee on the Safety of Nuclear Installations’ is followed [23]. It is taken as our ‘working definition’. Broadbent gives the following rather complete definition (quote) [23].

“The safety culture of an organization is the result of the interaction of individual and group values, attitudes, perceptions, skills, and behaviour that determine the commitment to, and the visible part and competency of, an organization’s safety performance. Organizations with a positive safety culture are characterized by communication founded on mutual trust, shared perceptions of the importance of safety, and confidence in the efficacy of preventive measures.”

In the scope of post-combustion CCS facilities, it is essential to understand the cognitive function, decision-making, and behaviours of individuals concerning safety protocols. Identifying the hazards when handling CO2 and chemicals, knowing the risks of failures, and following safety procedures are essential. In CCS, workers and managers need to prioritize safety when making decisions on operational procedures, equipment maintenance, and emergency responses. Engaging in positive safety practices can lower the chances of accidents, injuries, and environmental incidents.

### 2.2 System safety and practice

The concept of system safety originated in the missile production industry in the late 1940s [8]. Before the 1940s, system designers and engineers primarily used a trial-and-error approach to create safe designs [8]. This method was moderately effective during a time when system complexity was less compared to later advancements. This paper identifies parameters defining safety culture and explores their significance and how these parameters impact unplanned incidents.

Organisational culture is a surrogate for less visible facets of socio-technical systems, and its manifestation in safety performance [24]. The goal is to find ways and means of managing safety culture to avoid performance deficiencies and produce desirable outcomes. This view requires learning what should be changed to obtain the desirable behaviour. This paper employs a system dynamics model to answer these questions.

The visible part of an organisation is its physical configuration and hierarchies, procedures, and practices to achieve the organisational goals, that is safely producing for profit to survive, and also forging behaviours in line with the organisational goals. The visible part is how hierarchy, equipment and employees are organised and utilized to perform functions, with behaviours that are considered normal, which include the optimal level of competencies, skill mix, staffing, practices, procedures, and communication.

Reasoning that is employed in an organisation, includes beliefs, values, and practices that are used to keep the system functioning. The operators' culture is usually summed up in “this is the way we around here” and its justifications. Stories are constructed to relate to newcomers as to how they should behave. There are often values such as non-verbalised shared assumptions and unconscious expectations that underpin behaviours. These values are handed down from the old workforce to the new ones and reinforced by the management actions and policies as well as the company's code of conduct. These are drilled into employees and hence become part of their habits which are difficult to modify, as they are continuously reinforced through daily interactions and become the accepted practice.

Other cultural behaviours have their roots or are shaped externally, perhaps by the company's policy, work environment, hierarchy, and reward systems, which influence employees' decision-making. As the workforce moves between companies these behaviours may become globally almost uniform. However, primary aspects of common thinking may be tined by nationality, ethnicity, or religious beliefs. Thus, Organisational culture governs how the organisational tasks are ordered and done, and how behaviours are explained away and justified—i.e. narratives are weaved to justify how is done and why. Altogether these became the determinants of rationing attention regarding operational safety.

Organizations are varied in hierarchies, compartmentalised by disciplines, and diverse by ethnicity. Workforce moves between companies and even countries, thus some behavioural traits might be transferred from one job to the next, while others may be shared only in subgroups. The boundaries between subgroups are drawn as professional groups, and they compete for status, attention, and resources. The problems are further complicated due to the formation of sub-subgroups based on ethnicity, and so on. Some sub-subgroups may promote and enact practices that promote organisational goals, while others may undermine changes cascading down from the top.

The behaviour of engineers and managers can be different in many significant ways. For instance, engineers generally concentrate on running the system smoothly and safely, whereas managers may be more concerned with the company's bottom line. These focuses would have significant implications regarding priorities, especially for supervisors who must adhere to their subgroup or adopt the requirements of their new role as implementors of management priorities.

### 2.3 Safety Culture's influencing factors

Ibrahim Shire et al. have presented an extensive literature review about safety concerns using SD [25]. They have identified five themes: external factors, organisational influences, unsafe supervision, unsafe acts, and conditions for unsafe acts. They assert that SD can be used to improve safety performance. Churruca et al. have studied the dimensions of safety cultures using qualitative and quantitative methods [24]. They have identified 11 themes of safety culture. Zhang et al. used SD to study the role of risk perception chemical incident prevention when diverse groups of people are involved [26].

The emphasis on safety culture emerges from the objective of preventing significant disasters that could greatly affect a company, as well as minor yet disturbing incidents caused by everyday operations. Large multinational corporations attempt to minimize their vulnerability to losses by promoting a uniform global 'safety culture,' which may occasionally clash with their financial objectives. In this paper, the study utilized the five dimensions of safety culture outlined by Tappura et al. as the main dimensions for their analysis [27]. The study expands on previous research on safety culture, offering insight into its development within organizations. Most previous safety models assessed organizational safety culture maturity through surveys. The paper identified the key dimensions of safety culture based on their analysis as follows [27].

1. Commitment from management and supervisors,
2. Effective communication,
3. Organizational learning,
4. Training initiatives,
5. Employee dedication

These five key dimensions of safety culture are critical in the context of carbon capture and storage (CCS) facilities. Establishing a strong management commitment to safety is essential in determining the foundation for safety priorities and ensuring that safety is incorporated into all aspects of CCS operations. Encouraging employee involvement in safety decision-making and practices within the CCS facility develops a sense of ownership and responsibility, which in turn encourages their commitment to safety. Effective communication pathways enable quick responses to incidents, improve reliability, and adopt trust among employees, management, and local communities near CCS facilities. Comprehensive safety training and experienced personnel gaining their experience over time enable risk identification, hazard mitigation, and effective emergency response, ensuring safe CCS operations and environmental protection. Similarly, thorough safety training and investment in safety equipment, the identification of risks, the mitigation of hazards, and the implementation of effective emergency responses will develop gradually. This ensures the safety of CCS operations and environmental protection.

Risk communication and accident prevention are influenced by how individuals perceive risks, which are shaped by their experiences and judgment.

Dynamic risk assessment (DRA) serves as a framework that assists in making decisions in rapidly evolving and dynamic environments [28]. The characteristics of DRA include [29].

• Unforeseeable uncertainties and unexpected risks
• Situations characterized by rapidly changing risk environment.
• Changing and conflicting objectives
• Dynamic and evolving conditions that demand constant reassessment.
• High-risk levels
• Making fast decisions based on incomplete or inaccurate information.
• Employees required for on-site risk assessment.

Systems dynamics can help to understand this process by creating causal feedback loops in how events are mentally processed.

Like the lack of consensus in the definition of safety culture, there is no agreement regarding factors influencing its outcome. Several overlapping combinations of dimensions can be found in the literature. Such combinations of dimensions are used in this paper to build an SD model.

Safety culture in the literature is described using three or more of the following variables.

• Management commitment to safety.
• Worker's commitment to safety.
• Unrestricted communication (based on trust and no blame) at all levels.
• organizational learning (lesson learned).
• Thorough Hazard identification
• Reporting, and recording every hazard and near-misses.
• Willingness to use adequate expertise and financial resources.
• Shared belief, gaols, and common language (no ambiguity).
• Safety priority

Our primary goal is to study the safety culture impact on the safety outcome of a typical post-combustion carbon capture facility. We have identified primary variables that have the greatest influence on the safety performances. The model loses its usefulness if every variable is included.

### 2.4 System dynamics model

**Fig. 4:**
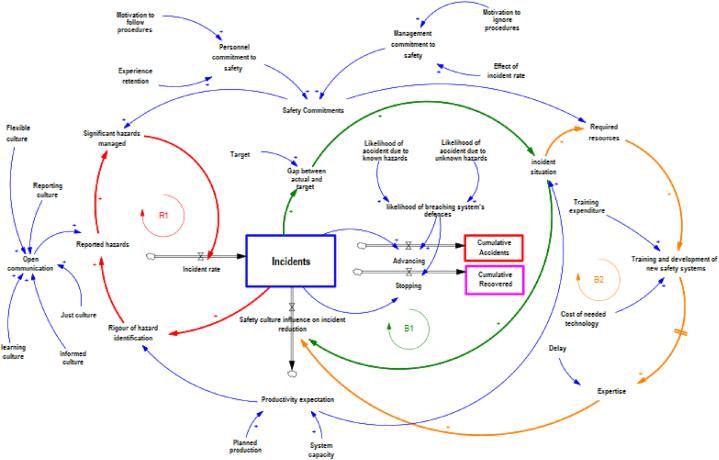
Effect of safety measures on the number of incidents using system dynamics.

System dynamics is an important tool in making decisions and analysing the behaviour of complex systems, as it aids in comprehending the outcomes of various choices and planning strategies for enhanced performance. Addressing issues through system dynamics requires a systematic process of following, modelling, and simulating the dynamic interactions within a complex system. Fig. 4 depicts the interactions between safety performance, safety measures, and accident rates within a gas and carbon system utilizing system dynamics.

Safety culture relies heavily on safety-related knowledge, emphasizing the importance of employee awareness, skill proficiency, targeted training, and education in comprehending safety protocols, demonstrating competence, and adhering to regulations [30]. Safety culture includes but extends beyond individuals’ attitudes and beliefs, incorporating elements like forward-thinking, shared accountability, efficient communication, ongoing safety training, preventative risk mitigation, and the implementation of safety procedures across all organizational hierarchies [30]. Workers frequently encounter a dilemma when balancing production goals, quality control, and compliance with safety regulations [31]. Achieving a middle ground between productivity and safety is a pivotal goal for the designers and managers overseeing safety-critical production systems [32]. The daily demands to boost production in a fiercely competitive environment often can lead to a shift in priorities, unconsciously choosing production over safety [33]. In the model, this term is called productivity expectation.

The reinforcing loop R1, as illustrated in Fig. 4, emphasizes the potential increase in risk factors. An incident may lead to a decline in trust in the current safety measures, which in turn can impact the motive to follow them, consequently undermining the safety management system. This can lead to insufficient accident documentation and the subsequent failure to address safety issues effectively thus resulting in increased risk levels. As the risk of incidents increases so do the frequency of incidents. Furthermore, the increase in management attention to incident situations can enhance the safety culture influence on incident reduction within the organization as shown in loop B1 in Fig. 4. When the difference between the current incident levels and the tolerable incident target is significant in loop B2 in Fig. 4. Organizations typically prioritize and allocate additional resources to address the problem. Under intense production pressure, safety focus may be compromised since only a limited management time would be available for addressing safety concerns. Adequate allocation of resources is essential for improving safety measures through increased targeted training and safety equipment investment. Availability of appropriate technology is crucial for the development of a new safety system. Eventually, these will improve safety culture and reduce incident rates. However, building a safety system is a time-intensive process with delayed outcomes.

This article uses a fictional installation with the trading name of ‘Invictus Carbon Recovery’, which is a carbon capture and utilization holding enterprise which has a few facilities and functions with a capture capacity of about forty tons of CO2 per day per facility. The company is assumed to experience sixty incidents per year, and the study aims to investigate the progression of incidents within the company by analyzing specific numerical factors over a 25-year projection. This analysis is based on a starting point of 60 incidents per year, termed as the reference case in the simulation. Table 2 shows the relationship between linguistic variables and numerical values in the SD model.

**Table 2:** Numerical values of constants.

Variable	Linguistic definition	Numerical definition	Reference condition
Cost of needed technology (on a scale of 0–1)	Low	0–0.25	0.6
	Medium	0.26–0.50	
	High	0.51–0.75	
	Very high	0.76–1.0	
Training expenditure (on a scale of 0–1)	Low	0–0.25	0.40
	Medium	0.26–0.50	
	High	0.51–0.75	
	Very high	0.76–1.0	
Two constants for management commitment to safety 1-Motivation to ignore procedures 2-Effect of incident rate (on a scale of 0–1)	Poor	0–0.25	1 = 0.80 2 = 0.75
	Average	0.26–0.50	
	Good	0.51–0.75	
	Excellent	0.76–1.00	
Two constants for personnel commitment to safety 1-Motivation to follow procedures. 2-Experience retention. (on a scale of 0–1)	Poor	0–0.25	First = 0.80 Second = 0.7
	Average	0.26–0.50	
	Good	0.51–0.75	
	Excellent	0.76–1.00	
Open Communication- an average of ‘Just culture’, ‘Reporting culture’, ‘Learning culture’, and ‘Flexible culture’, (Fig. 3). (all on a scale of 0–1)	Weak	0.00–0.35	All set at 0.5
	Average	0.36–0.70	
	Excellent	0.71–1.00	
Likelihood of accident due to known hazards	From the safety case reports	Must be less than 5*10^−4^	0.0005
Likelihood of accident due to unknown hazards	Estimated	One-tenth of known hazards	0.00005
Planned production (as a fraction of the capacity) (on a scale of 0.1–0.2)	May not break even	0.1–0.3	1
	Usual workload	0.4–0.6	
	High	0.7–0.8	
	Extreme	0.9–1	

**Fig. 5:**
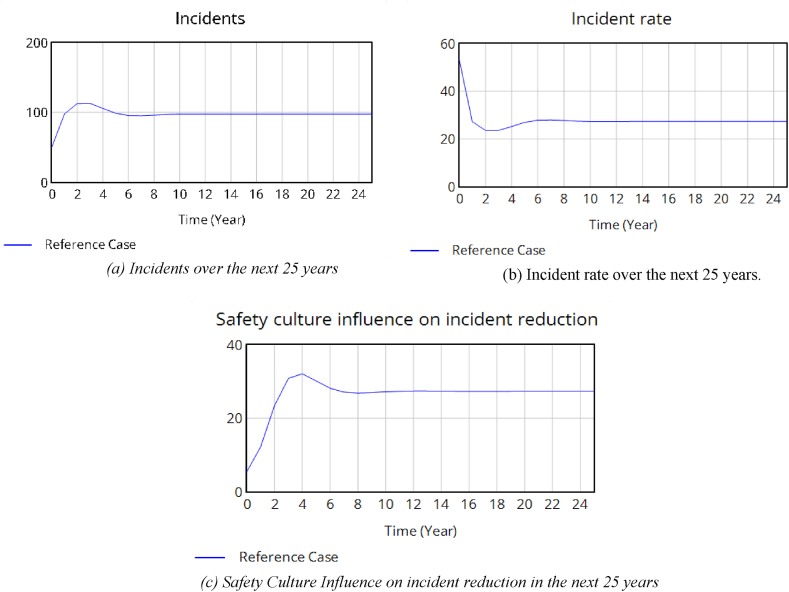
(a) Incidents over the next 25 years (b) Incident rate over the next 25 years. (c) Safety Culture Influence on incident reduction in the next 25 years.

In the model, the annual number of incidents is calculated as the difference between the incident rate and safety culture influence on incident reduction, as illustrated in Fig. 5(a). The incident rate is indirectly dependent on safety commitment and current hazard level and directly dependent on significant hazards managed. Fig. 5(b) shows the incident rate in the next 25 years.

**Fig. 6:**
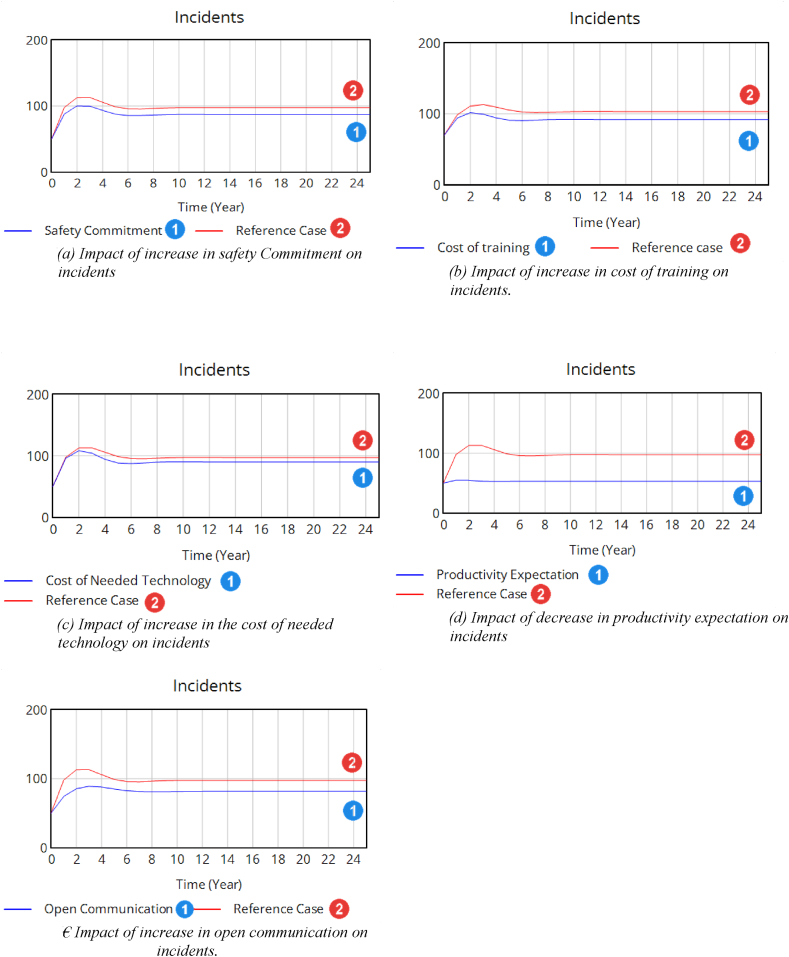
(a) Impact of increase in safety Commitment on incidents (b) Impact of increase in cost of training on incidents. (c) Impact of increase in the cost of needed technology on incidents (d) Impact of decrease in productivity expectation on incidents (e) Impact of increase in open communication on incidents.

The incidents that progress to near-mises or accidents, though must be avoided, but offer chances to learn from experience, improve procedures, and reduce the likelihood of unwanted events happening again [34]. The effect of safety culture on incident reduction is shown Fig. 5(c) over the next 25 years.

Given limited resources to mitigate occupational injuries, companies face challenges in determining the most effective allocation of these resources to achieve maximal injury reduction at an optimal cost. The way employees view the safety system is linked to the dedication of management to safety, which seems to impact the incident rates [35]. The study reveals that safety performance is enhanced by increased safety investments including training and cost of equipment, a stronger safety culture, or reduced project hazards. The impact of each factor on safety performance is influenced by variations in other factors, with safety culture playing a mediating role in the effect of voluntary safety investments [36]. Recent studies indicate that 20 % of accidents involve safety-critical communications (SCC) as a contributing factor [37]. Safety professionals can use available information to create communication strategies that promote a safety-oriented culture and address challenges in safety communication [38].

## 3 What-if analyses

### 3.1 Impact assessment of variables

An analysis of the impact of certain constant variables was conducted to uncover their internal connections. Five distinct scenarios were assessed to examine their influence on the frequency of incidents and to determine the variable with the most notable impact on incident numbers. These scenarios are detailed in Table 3.

**Table 3:** Studying various scenarios to assess how different factors affect the number of incidents.

Scenario	Values	Outcome
1. Safety commitments (function of two variables)	0.75 → 0.9	In this case, the minimum amount of both “management commitment to safety” and “personnel commitment to safety” will be the governing value. The number of incidents decreased from 97 to 88 incidents per year after 9 years as shown in Fig. 6(a).
2. Training expenditure (as an investment)	0.4 → 0.9	The number of incidents decreased from 97 to 87 incidents per year after 9 years as shown in Fig. 6(b).
3. Cost of needed technology (as an investment)	0.6 → 0.9	The number of incidents decreased from 97 to 91 incidents per year after 9 years as shown in Fig. 6(c).
4. Productivity expectation	10 → 5	The number of incidents decreased from 97 to 51 incidents per year after 9 years as shown in Fig. 6(d).
5. The value for ‘Open Communication’ is an average of four dimensions of culture as shown in Fig. 3.	0.5 → 0.9	The number of incidents decreased from 97 to 81 incidents per year after 9 years as shown in Fig. 6(e).

## 4 Discussion

According to the study done by Samadi and Garbolino [39], in the mid-1980s, disasters like Three Mile Island, Bhopal, Chernobyl, and Challenger shaped safety culture and resilience concepts in safety management [39]. These events emphasized the need to address organizational and management challenges in safety practices. Organizations can improve safety culture and performance by implementing strategies in various industries, including post-combustion carbon capture facilities. safety culture enables an organization through which to enhance safety performance. safety culture is considered as an acquired characteristic of an organization which manifests itself when the performing performs its function. This characterizes safety culture as a temporary state of an organization, that may change depending on operational or economic needs. Several safety researchers have attempted to identify primary variables that govern safety culture. A consensus among these researchers is to ensure high safety performance management should focus on safety culture as opposed to focusing solely on the management of hazards and their consequences.

Safety culture research is now at a mature stage of development, and the link between safety culture and safety performance is well-established [40]. However, there were attempts to integrate the concept of safety culture into the systems-based simulation model. Such models can provide more insight into the impact of various safety culture's influencing factors for a given case. System dynamics is such a tool that can assist in shaping policies for an effective safety culture in an organization.

In today's competitive business landscape, one of the primary obstacles for organizations is establishing and maintaining a self-sustaining safety culture [41]. Indicators of an inadequate safety culture include:

Lack of open communication: In many cases, small incidents or near-misses are overlooked until a major accident or injury happens. This can be due to a lack of reporting, inadequate safety measures, or a failure to recognize warning signs.

High productivity expectations: The organization prioritizes profitability above all else, viewing health and safety as an expense to be minimized to avoid additional costs. Increased productivity expectations can contribute to a higher incidence of workplace incidents by raising stress levels, shifting attention from safety procedures to task completion, and reducing oversight of safety protocols by supervisors who are focused on meeting productivity targets.

Insufficient safety investment: The organization does not allocate enough resources including training and technology financing to promote safety culture. This can be due to cost concerns, lack of awareness, focus on short-term results, and lack of adequate support.

Lack of safety commitment: Inadequate support from management and engagement from employees in safety initiatives can encourage an environment where safety is not prioritized, putting workplace safety at risk. the need to achieve more with fewer resources may lead management to make decisions that compromise safety standards.

All these factors are key factors that impact the safety culture. When the behaviours of these elements are understood, suitable policies for promoting and maintaining a safe work environment may be devised. Alshehri et al. stated employee behaviours, management attitude, and the working environment are three primary dimensions of the safety culture [42]. Of these three, they believe management policy, i.e. management behaviour, safety policy enforcement, and a safety management system, are the main contributors [42].

It's important to recognize that safety culture can differ widely across regions due to various factors like local regulations, cultural norms, historical incidents, and industry practices as well as the ethnic mix of operators, social grouping and so on. These variations impact attitudes toward safety, perceptions of risk, communication styles, and overall approaches to safety management in different regions. Also, the factors influencing safety performance are numerous and extend beyond the parameters outlined in the paper. It is crucial to consider additional variables and their impact on safety culture in future research studies.

## 5 Conclusions

Understanding the interplay of components of complex systems can be a challenging task. SD is a simulation technique reliant on computers, mainly used for examining evolving complex systems, both qualitatively and quantitatively. To analyse risks and hazards using system dynamics, a structured approach is needed to observe relationships within the system, build models, and simulate the dynamic relationships of the system's elements. In this study, the system dynamics approach is employed to analyse factors influencing safety culture and hence performance. The SD approach is a suitable method to study safety issues since it enables a comprehensive insight into the behaviour of interconnected components that affect safety outcomes. The study's results indicate that the frequency of incidents is notably impacted by expectations around productivity. Furthermore, factors like safety investment, open communication, and safety commitment also exert varying degrees of influence on incident rates using the system dynamics approach. Organizations can improve safety culture and performance in the long run by examining the interconnections among different factors that impact safety outcomes, allowing them to make well-informed decisions.

## CRediT authorship contribution statement

**Maryam Shourideh:** Writing – original draft. **Sirous Yasseri:** Writing – review & editing, Validation, Supervision. **Hamid Bahai:** Validation, Supervision.

## Declaration of competing interest

The authors declare that they have no known competing financial interests or personal relationships that could have appeared to influence the work reported in this paper.

## Figures and Tables

**Figure:**
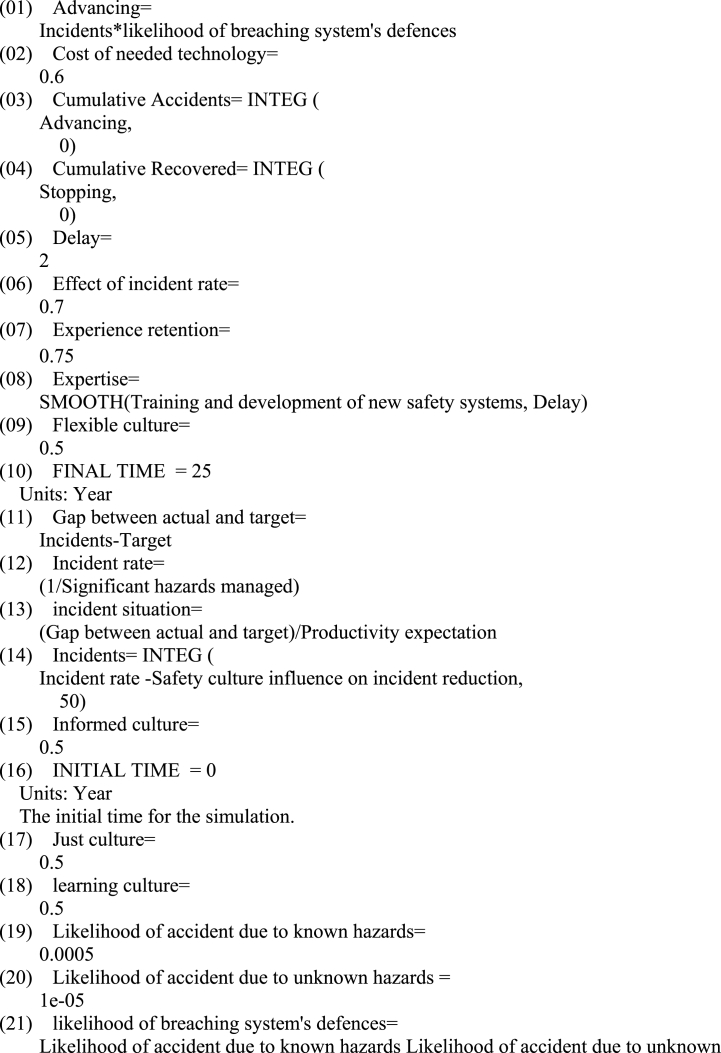

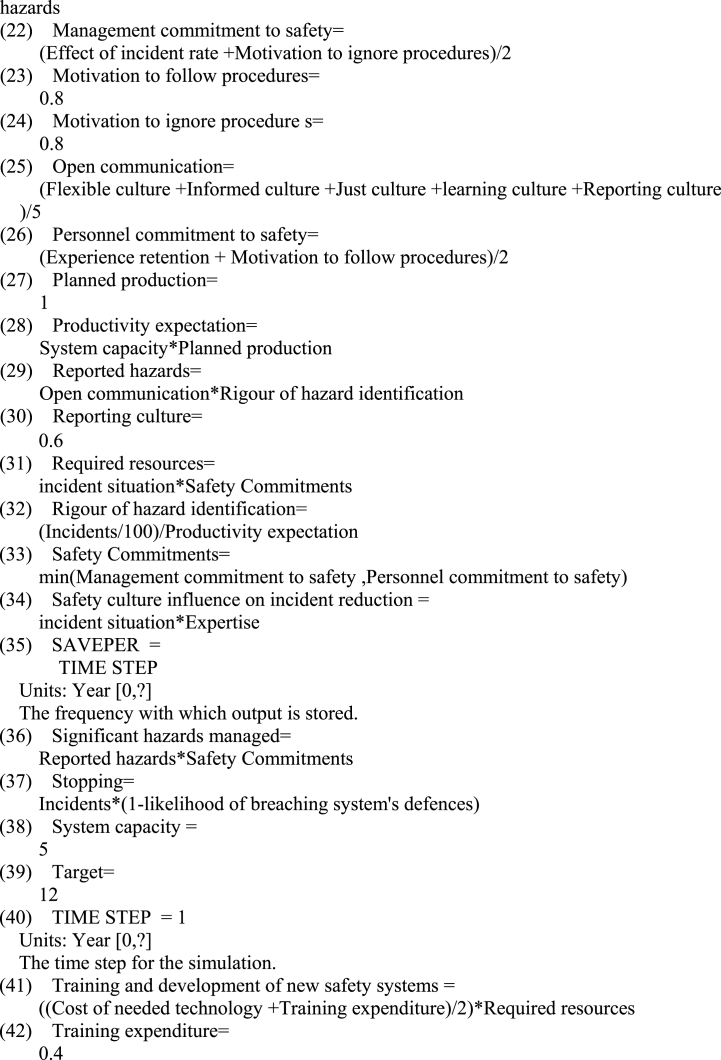


## Footnotes

1 Incident = An event that has unintentionally initiated, but it has not advanced enough to become an accident. This could be due to the incident running out of energy, sheer luck or being arrested by the system defences.

2 Accident = An incident which has progressed breached the system defences, and could potentially, but not necessarily, cause harm.
